# Comprehensive Analytical Interceptive Approach to Manage Crowding in Mixed Dentition: A Case Report

**DOI:** 10.7759/cureus.93128

**Published:** 2025-09-24

**Authors:** Himani Gupta, Divya S Sharma

**Affiliations:** 1 Department of Pediatric and Preventive Dentistry, Dr. Ziauddin Ahmad Dental College and Hospital, Aligarh Muslim University, Aligarh, IND

**Keywords:** guidance of eruption, incisor crowding, interceptive orthodontics, mixed dentition, mixed dentition analysis, space supervision

## Abstract

Dental crowding is among the most prevalent forms of orthodontic malocclusion, commonly observed during the mixed dentition period, with the mandibular anterior region being the most frequently affected. The management of crowding during the early to mid-mixed dentition period has been described using various terms, including space supervision, eruption guidance, pre-orthodontic guidance, and interceptive orthodontics. An analytical approach from extraoral to intraoral examination, along with a mixed dentition model analysis, is vital for effective space supervision, as it helps determine the space needs, thereby aligning the erupting permanent teeth.Space supervisionis a broader term for space maintenance and preservation, maxillary expansion, disking of primary teeth, selective extraction of primary teeth, and minor tooth movements to preserve the arch dimensions and facilitate oral functions. This case report presents the diagnosis and management of a mixed dentition malocclusion case of a 10-year-old female patient with severe maxillary and mandibular incisor crowding along with anterior crossbite with a comprehensive analytical interceptive non-extraction approach sequentially applied through space supervision, including maxillary expansion, disking of primary canines, 2×4 appliance therapy, and conservation of leeway space by lingual holding arch.

## Introduction

The World Health Organization (WHO) recognizes malocclusion as one of the most significant oral health issues, ranking it after dental caries and periodontal disease [1]. Dental crowding is the most prevalent form of orthodontic malocclusion, commonly observed during the mixed dentition, in the mandibular anterior region [2]. The severity of crowding is assessed using tooth size-arch length discrepancy (TSALD) with the help of mixed dentition analysis. The American Academy of Pediatric Dentistry recommends that the arch length and crowding must be considered in the context of the esthetic, dental, skeletal, and soft tissue relationships [3]. Dental crowding is affected by both genetic and environmental influences, including insufficient space in the dental arch, large permanent teeth, premature loss or prolonged retention of primary teeth, hormonal imbalances, and the presence of supernumerary teeth, tumors, or cysts, among others [4]. 

Dental crowding in mixed dentition negatively affects esthetics, occlusion, mandibular-cranial relation, and oral hygiene maintenance. The prevalence of dental crowding among children and adolescents is 56%, ranging from 31% to 96% depending on the geographic location [5]. A systematic review showed the prevalence of crowding in mixed dentition to be 11.8% in Asia and 37% worldwide, which increased to 50% and 39%, respectively, in permanent dentition [6].

On the basis of TSALD, dental crowding is classified as mild (1-3 mm), moderate (3-5 mm), and severe (>5 mm) [5]. Lower anterior crowding is considered normal, with an average discrepancy of -1.6±1.0 mm following the eruption of the permanent incisors. Most children between the ages of eight and nine exhibit 0-3.5 mm lower incisor crowding [7].

Mixed dentition analysis for orthodontic diagnosis and treatment planning of developing malocclusions in children helps determine whether the treatment will involve simple monitoring, eruption guidance, space regaining, space maintenance, or serial extractions [8]. Various clinically applicable mixed dentition analyses can be done using the Ortho Assistant© web application that provides a detailed yet simplified guide for quick and accurate calculations for most of the spatial dimensions in the dental arch. Calculations to derive the most commonly used TSALD (brass wire or segmental arch analysis) and transverse analysis (Ponts and Schwarz analysis) are available in this web application [9]. This application also takes into account the Class I correction in the mandible for the calculation of TSALD in cases with end-on posterior occlusion. Two unique clinically relevant terms, that is, incisor space discrepancy (ISD) and buccal space discrepancy (BSD), in this application, referred to the discrepancy between the incisor/buccal space on both sides and the combined mesiodistal widths of the respective permanent incisors and buccal succedaneous teeth [9]. The term "ISD" effectively calculates the extent of crowding in the anterior region during the transitional phase, as the permanent incisors are available most of the time in early mixed dentition, when early interception is most effective. 

Following the comprehensive spatial analysis, the management of crowding in the mixed dentition period can be done with the treatment procedures that affect the eruption patterns and alignment of permanent teeth during the transition from primary to mixed dentition, commonly termed as eruption guidance, pre-orthodontic guidance, and interceptive orthodontics [10].

Space supervision and eruption guidance during the mixed dentition are suitable for moderate or mild TSALD, <5 mm in the maxillary arch and <3 mm in the mandibular arch, with the deficiency incorporating any available bilateral leeway space [11]. Incisor crowding during the mixed dentition is resolved using methods like interproximal reduction or disking of primary canines, maxillary expansion, proclination of the permanent incisors, and extraction of primary canines (planned serial extraction). Bell and Sonis [10] described an effective technique of creating a "sluiceway" by reducing the mesiolingual corners of the primary canines and allowing lingually displaced incisors to move forward under the influence of tongue pressure that preserves or increases arch circumference. This creates a 1-2 mm space per side by facilitating the unraveling of crowded mandibular incisors labially without or mildly encroaching the leeway space [10]. The other approach to gain space is by increasing the transverse dimensions of the arch by expansion using a removable or fixed appliance. The 2×4 fixed appliance, being commonly used during the early mixed dentition period for incisor alignment, is a very versatile, comfortable, easy-to-use, and well-tolerated appliance [12]. This study presents a case report of a comprehensive analytical interceptive approach for the diagnosis and interceptive management of severe crowding in mixed dentition, utilizing a combination of interventions such as maxillary expansion, selective disking of the primary canines, and fixed 2×4 appliance therapy followed by lingual holding arch.

## Case presentation

**Figure 1:**
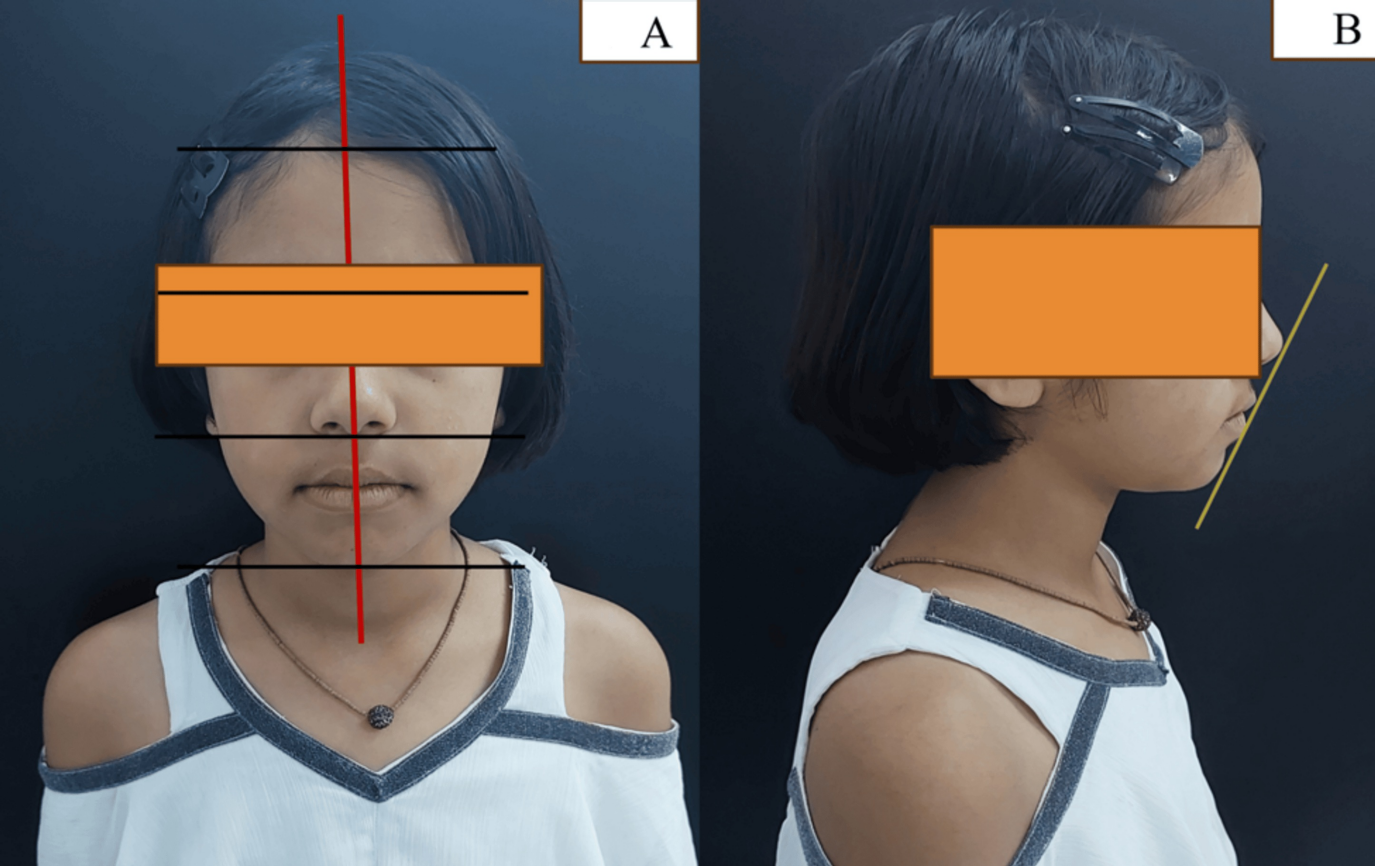
Extraoral clinical images (A) Front profile showing a bilaterally symmetrical face. (B) Side profile showing a straight facial profile with competent lips and S line showing a well-balanced face with the upper and lower lips touching the line

A 10-year-old female patient with a chief complaint of malaligned teeth presented to the Department of Pediatric and Preventive Dentistry. There was no significant medical history, but dental history included a recent tooth extraction in the upper left posterior region. The patient had a normal posture and build. Extraoral examination revealed a bilaterally symmetrical face, straight facial profile, and competent lips (Figure 1A-1B).

**Figure 2:**
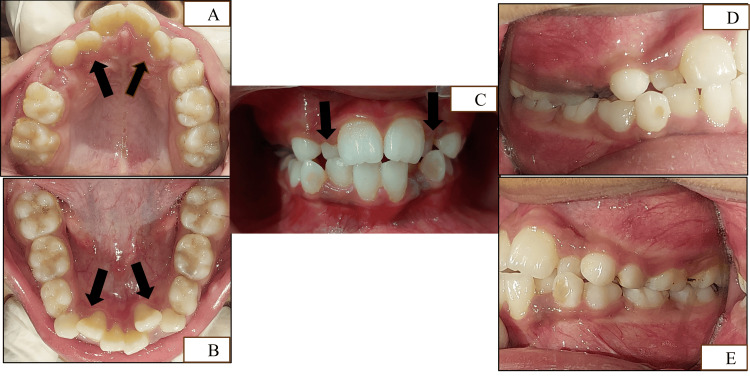
Intraoral clinical images (A) Maxillary occlusal view (arrows pointing towards the crowded anterior region). (B) Mandibular occlusal view (arrows pointing towards the crowded anterior region). (C) Front occlusal view (arrows pointing towards the teeth in crossbite). (D) Right side occlusion view. (E) Left side occlusion view

Intraoral examination revealed a constricted maxillary arch with crowded maxillary and mandibular incisor regions (Figure 2A-2E).

**Figure 3:**
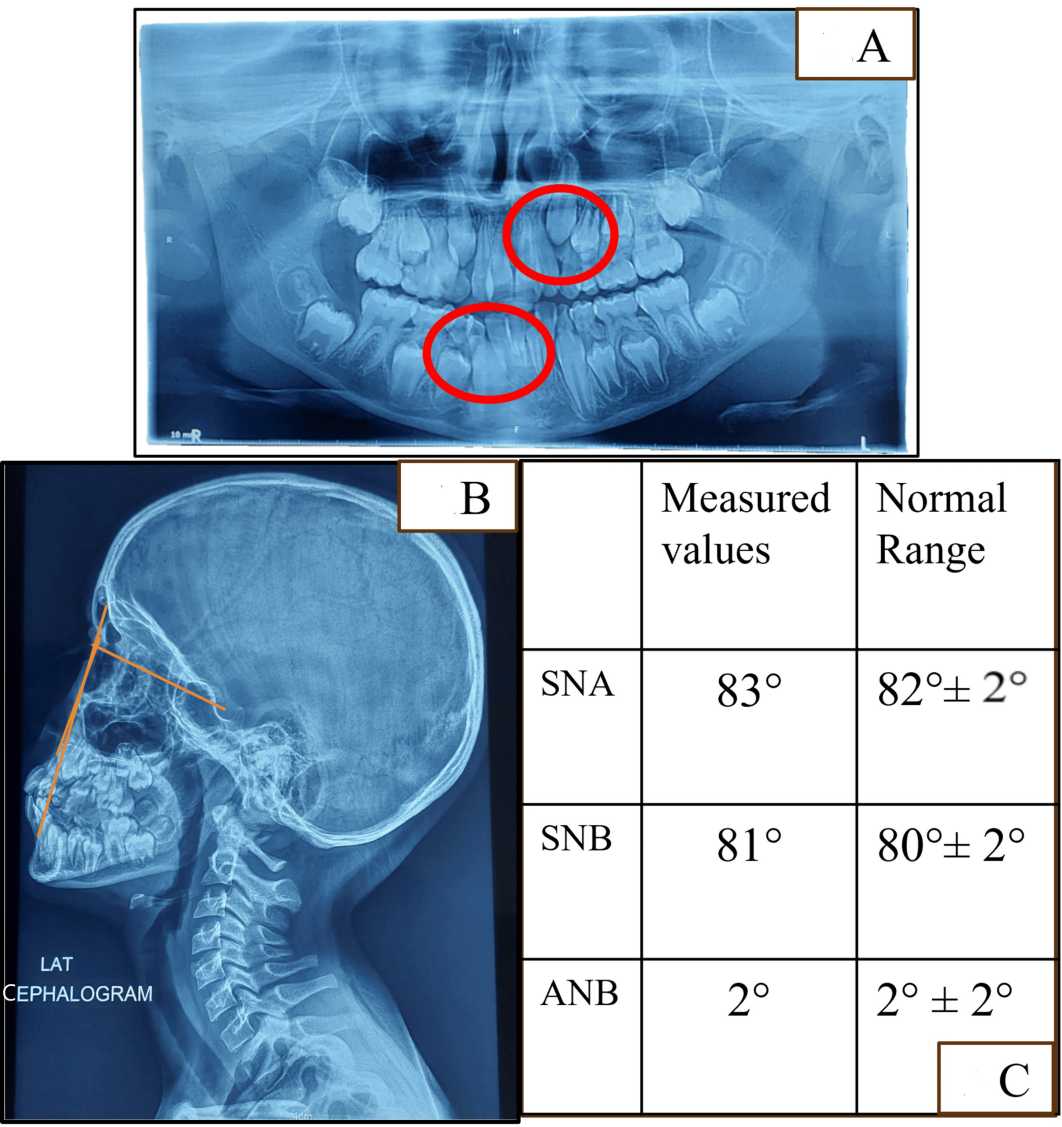
Radiographic examination (A) OPG revealing an overall lack of space for the succedaneous teeth. (B) SNA, SNB, and ANB markings on the cephalogram. (C) SNA, SNB, and ANB values within the normal range OPG: orthopantomogram; SNA: angle between the sella, nasion, and subspinale point A; SNB: angle between the sella, nasion, and supramentale point B; ANB: angle between the subspinale point A, nasion, and supramentale point B

There were lingually erupting #12, #22, #32, and #42 and missing #54. Developing crossbite with respect to #12, #22, and #53 was evident along with a midline shift towards the right. Angle's Class I was present on both sides (Figure 2D-2E). Functional examination revealed nasal breathing, a mature swallowing pattern, and bilateral chewing with synchronous temporomandibular joint (TMJ) movements. Functional mandibular shift was not found, and there were no premature contacts during mandibular closure, confirming that the midline deviation was due to dental reasons. There was no history of oral habits. Orthopantomogram (OPG) suggested space deficiency for the erupting permanent canines with an overall shortage of space for the succedaneous teeth in all four quadrants (Figure 3A).

**Figure 4:**
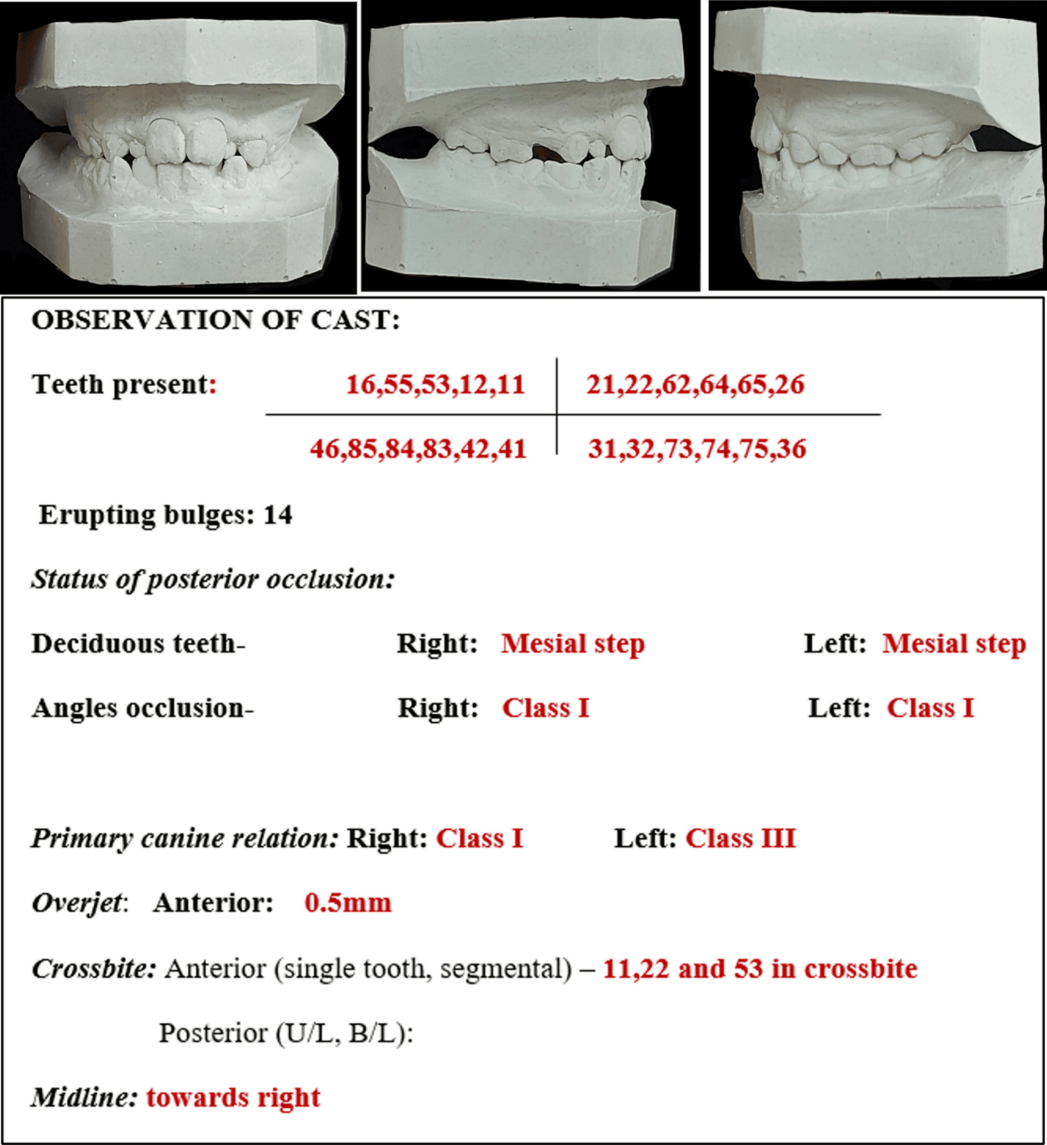
Study models of the patient in frontal and side occlusal views along with the detailed observations from the cast

Lateral cephalogram was used to do Steiner's analysis that revealed SNA (angle between the sella, nasion, and subspinale point A) and SNB (angle between the sella, nasion, and supramentale point B) in the normal range, at 83 and 81 degrees, respectively (Figure 3B-3C). Impression making of maxillary and mandibular arches, followed by model fabrication, was done for detailed observation (Figure 4) and mixed dentition analysis.

Mixed dentition model analysis using Tanaka and Johnston was done using the Ortho Assistant© web application [9]. Segmental analysis (Table 1) revealed an ISD of -7 mm and -5 mm in the maxillary and mandibular arches, respectively. Combined (right and left) BSD of -1 mm and +2 mm was found in the maxilla and mandible, respectively. Thus, TSALD (using Tanaka and Johnston) was found to be -8 mm in the maxillary arch and -3 mm in the mandibular arch.

**Table 1:** Segmental analysis (incisor space discrepancy and buccal space discrepancy) and TSALD TSALD: tooth size-arch length discrepancy

	Maxilla	Mandible
Incisor space discrepancy (mm)	-7	-5
Buccal space discrepancy (mm)	-1	2
All total discrepancy (TSALD) (mm)	-8	-3

This discrepancy reveals severe crowding in the maxillary arch and moderate crowding in the mandibular arch. Transverse arch analysis using Schwarz analysis revealed the calculated premolar/molar value (CPV/CMV) to be more than the measured premolar/molar value (MPV/MMV) (Table 2) in both maxilla and mandible, and the Korkhaus palatal index (45.61) revealed a high palate, both indicating the need for arch expansion. 

**Table 2:** Schwarz transverse analysis values (calculated values are greater than the measured values indicating the need for expansion)

Schwarz analysis	Maxilla	Mandible
Measured premolar value (mm)	31	33
Calculated premolar value (mm)	43	43
Discrepancy	-10	-8
Measured molar value (mm)	45	46
Calculated molar value (mm)	50	50
Discrepancy	-5	-4

Following these investigations, a comprehensive treatment plan was formulated which included the expansion of the maxillary arch using a removable appliance incorporating jackscrew, sluiceway preparation in maxillary and mandibular primary canines to allow the labial proclination of lateral incisors along the slopes of the canines, 2×4 appliance therapy for the proper alignment of the incisors along the arch form, and lingual holding arch placement after incisor correction to hold the labio-lingual position of incisors, as well as to preserve leeway space.

**Figure 5:**
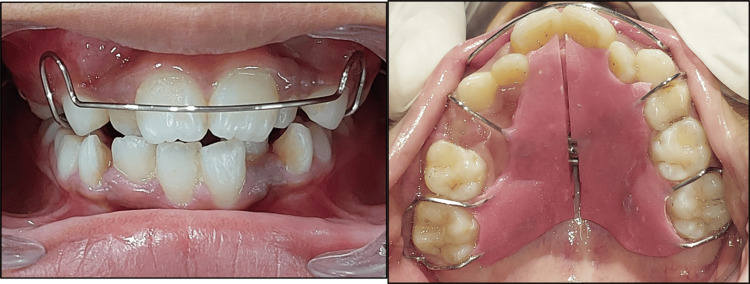
Removable appliance incorporating jackscrew

In the first phase of treatment, a removable expansion appliance with jackscrew (Figure 5) was delivered to the patient.

**Figure 6:**
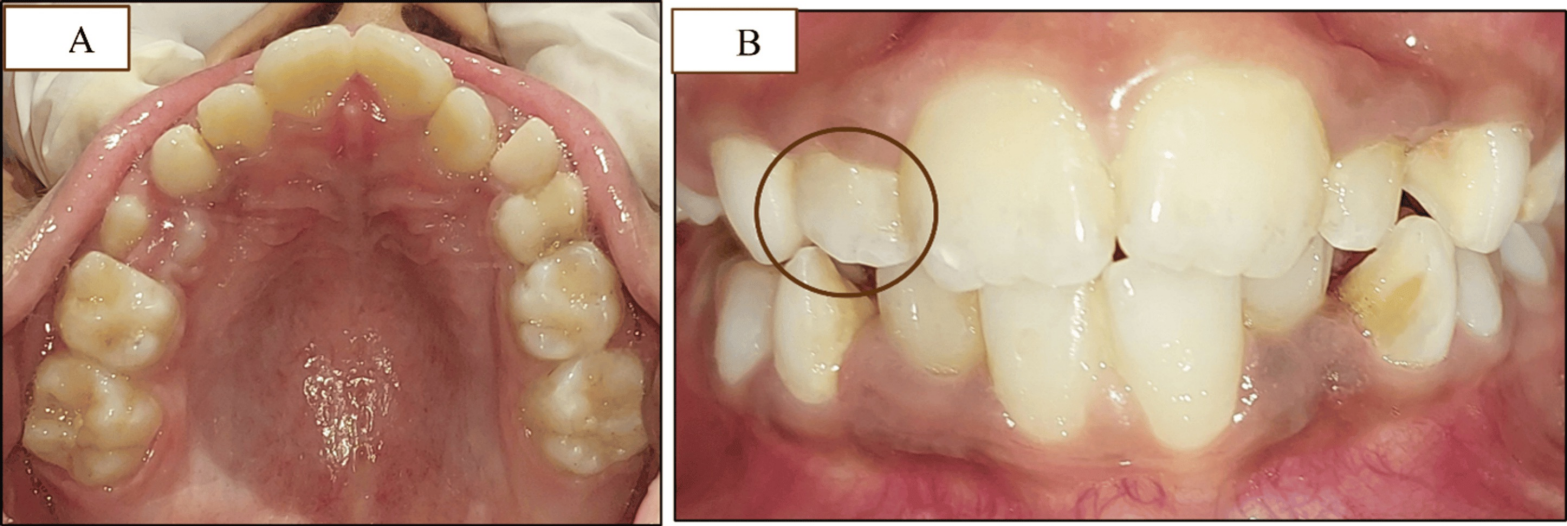
Follow-up images after removable expansion appliance therapy (A) Expanded maxillary arch. (B) Crossbite corrected with respect to #12 while crossbite with respect to #22 still evident

The patient was asked to activate the appliance four times a week, every other day. Postoperative instructions were given, and the patient was kept on weekly follow-ups. At the end of six weeks, the MPV and MMV increased by about 5 mm (Figure 6A).

**Figure 7:**
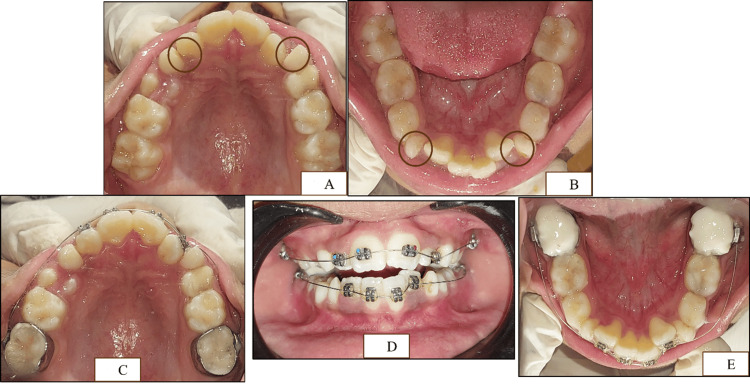
The second phase of treatment with sluiceway preparation and 2×4 appliance therapy (A) Sluiceway preparation on the maxillary primary canines. (B) Sluiceway preparation on the mandibular primary canines. (C) 2×4 appliance on the maxillary arch. (D) Frontal view of the 2×4 appliance. (E) 2×4 appliance on the mandibular arch along with bite blocks of GIC on the first permanent mandibular molars GIC: glass ionomer cement

Crossbite with #12 and #53 was improved, but crossbite with #22 still remained (Figure 6B). In the next phase, sluiceway preparation was done in #53, #63, #73, and #83 (Figure 7A-7B), followed by a 2×4 appliance in the maxillary and mandibular arches.

**Figure 8:**
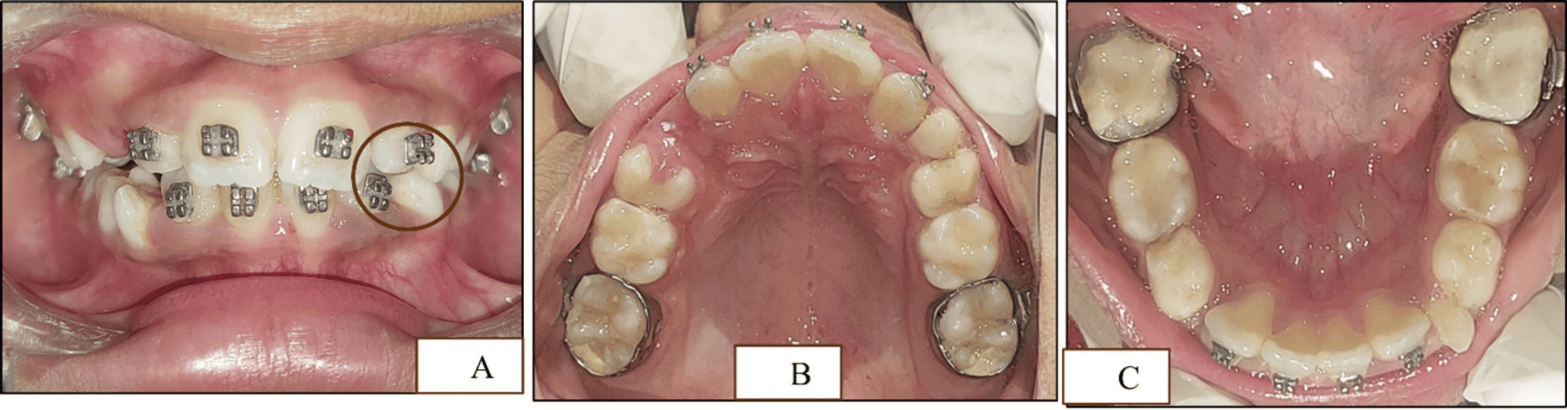
One-month follow-up after 2×4 appliance therapy (A) Crossbite corrected with respect to #22. (B) Slight labial proclination of the maxillary incisors, with #53 exfoliated and #14 erupted. (C) Labial movement of the mandibular lateral incisors with respect to #32 and #42, with #83 exfoliated

Preformed band adaptation was done on #16, #26, #36, and #46, followed by the placement of McLaughlin, Bennett, and Trevisi (MBT) brackets on all the permanent incisors along with a 0.012" round nickel-titanium (Ni-Ti) archwire. Bite was raised using a glass ionomer cement (GIC) block on the lower molars (Figure 7C-7E). On the one-month recall, crossbite with respect to #22, as well as the midline shift, was corrected (Figure 8A) along with a slight labial movement of #12 and #22 in the maxillary arch with the exfoliation of #53 and the eruption of #14 (Figure 8B).

**Figure 9:**
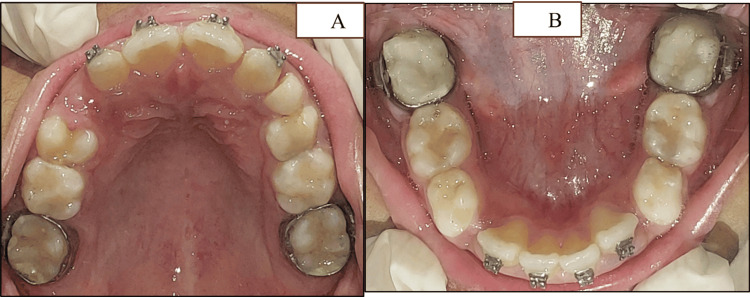
Three-month follow-up after 2×4 appliance therapy (A) Crowding relieved in the maxillary incisor region along with well-aligned maxillary incisors along the arch form. (B) Crowding relieved in the mandibular incisor region along with well-aligned mandibular incisors

The wire was changed to a 0.014" round NiTi archwire. At the second-month follow-up, the wire was changed to a 0.016" round NiTi archwire. At the three-month recall, #73 was exfoliated with well-aligned maxillary and mandibular teeth (Figure 9A-9B).

**Figure 10:**
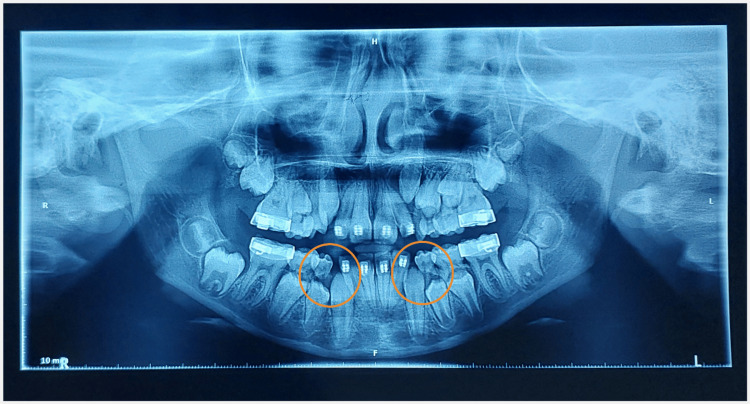
Three-month follow-up OPG after 2×4 appliance therapy revealing a lack of space for the erupting mandibular permanent canines OPG: orthopantomogram

OPG at this time revealed a lack of space for the erupting permanent canines (Figure 10). 

**Figure 11:**
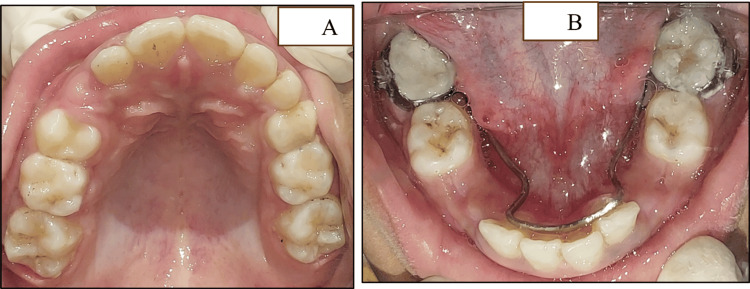
Clinical images of the maxillary and mandibular arches after 2×4 appliance removal (A) The 2×4 appliance removed with respect to the maxillary arch after the attainment of well-aligned dentition. (B) The 2×4 appliance removed with respect to the mandibular arch after the attainment of well-aligned dentition along with the extraction of the first primary mandibular molars and the placement of the lingual arch

The first primary mandibular molars were extracted to provide space for the erupting permanent canines. The 2×4 appliance was removed, and a lingual holding arch was placed (Figure 11A-11B).

Huckaba's analysis, done with the same Ortho Assistant© web app, revealed around a 2 mm excessive E-space compared to the respective second premolar, which would be utilized in the future at the time of the eruption of the first premolars after further assessment of the space need.

## Discussion

A thorough assessment of a malocclusion case during the mixed dentition phase should include both static and functional occlusion evaluation followed by radiographic and mixed dentition model analysis in all planes. The midline shifts in the mixed dentition may be purely dental, functional, or skeletal. It is important to rule out functional mandibular shift, as, if undetected, it can lead to the permanent teeth intercuspating into a shifted mandibular position, creating centric occlusion (CO)-centric relation (CR) discrepancy affecting facial asymmetry and TMJ issues [13].

Detection and interception of a developing malocclusion in the mixed dentition are recommended for crowding, which might worsen in the permanent dentition. Severe crowding is often associated with poor oral hygiene, gingivitis, gingival recession, dental caries, tooth mobility, and eventual tooth loss. Such conditions can impact an individual's functional abilities, emotional well-being, and social interactions [14].

There is no expected increase in intercanine width after the lower lateral incisor erupts [15]. For precarious space conditions (borderline arches) in the first transitional phase, maxillary intercanine width increase is dependent on Class I primary canine relation bilaterally during the erupting phase of the lower lateral incisors [16]. Incisor liability is overcome by the labial positioning of incisors and intercanine width increase in these cases, provided environmental factors, e.g., proximal caries or habits, are not affecting dental arches, which may result in rapid arch space loss. Reduction in arch length in the buccal segment and uprighting of the incisors in the second transition phase together contribute to a significant decrease in the arch perimeter [17]. This leads to worsening of the existing crowding within the mandibular arch. The model analyses in different planes and segments localize the targeted treatment plan to intercept developing crowding and take advantage of erupting dentition and adaptive muscular engram in growing children.

**Figure 12:**
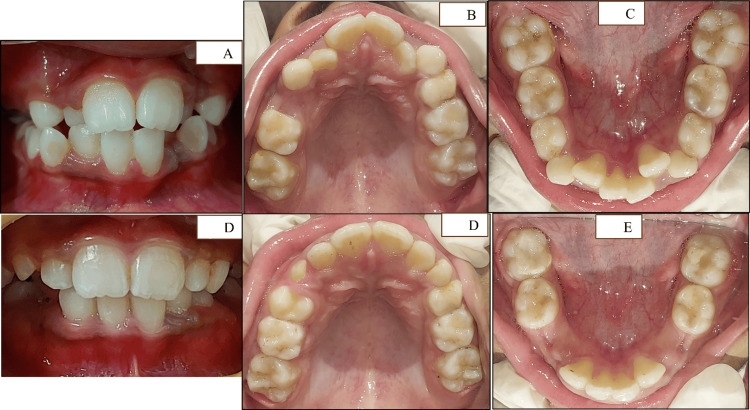
Pre- and postoperative clinical photographs after interceptive orthodontic treatment (A-C) Preoperative images. (D-E) Postoperative images

Our treatment plan followed the American Association of Pediatric Dentistry (AAPD) guideline to maintain the original arch form. Severe TSALD in the maxilla (-8 mm) and mandible (-5 mm) guided us to assess the transverse discrepancy in the premolar and molar regions. Schwarz analysis was preferred as it allows measurements in primary dentition and asks for the face type too. The Ortho Assistant© web app not only incorporates these variables but also guides for correct measurements. The -5 mm discrepancy in the premolar region was resolved with the help of expansion that partially resolved anterior crowding. The expanded arch facilitated the labial movement of incisors through the sluiceway created on the primary canines (Figure 12A-12E).

Posteriorly in the buccal segment, the BSD calculation predicted crowding in the maxilla. Often, at the time of the second transitional phase (buccal teeth exchange), the maxillary excess leeway space is used for incisor uprighting. However, negative BSD in the maxilla was resolved by an expansion appliance. In the mandible too, excess leeway space was maintained as no Class I correction was needed (late mesial shift) because molars were already in a properly intercuspated Class I relation. Thus, it was used for further alignment of mandibular incisors. A lingual holding arch was fixed in the mandible to prevent the mesial drift of the first permanent molars and the lingual tipping of lower incisors, thereby maintaining the arch perimeter. The first primary molars had to be extracted to timely distalize the erupting permanent canine, as OPG showed a lack of space for their eruption (Figure 10). Extractions or mesial reduction of primary teeth distal to erupting buccal succedaneous teeth require proper timing. At the time of the first premolar eruption, mesial disking of the second primary molar would align the buccal teeth and resolve BSD. 

**Figure 13:**
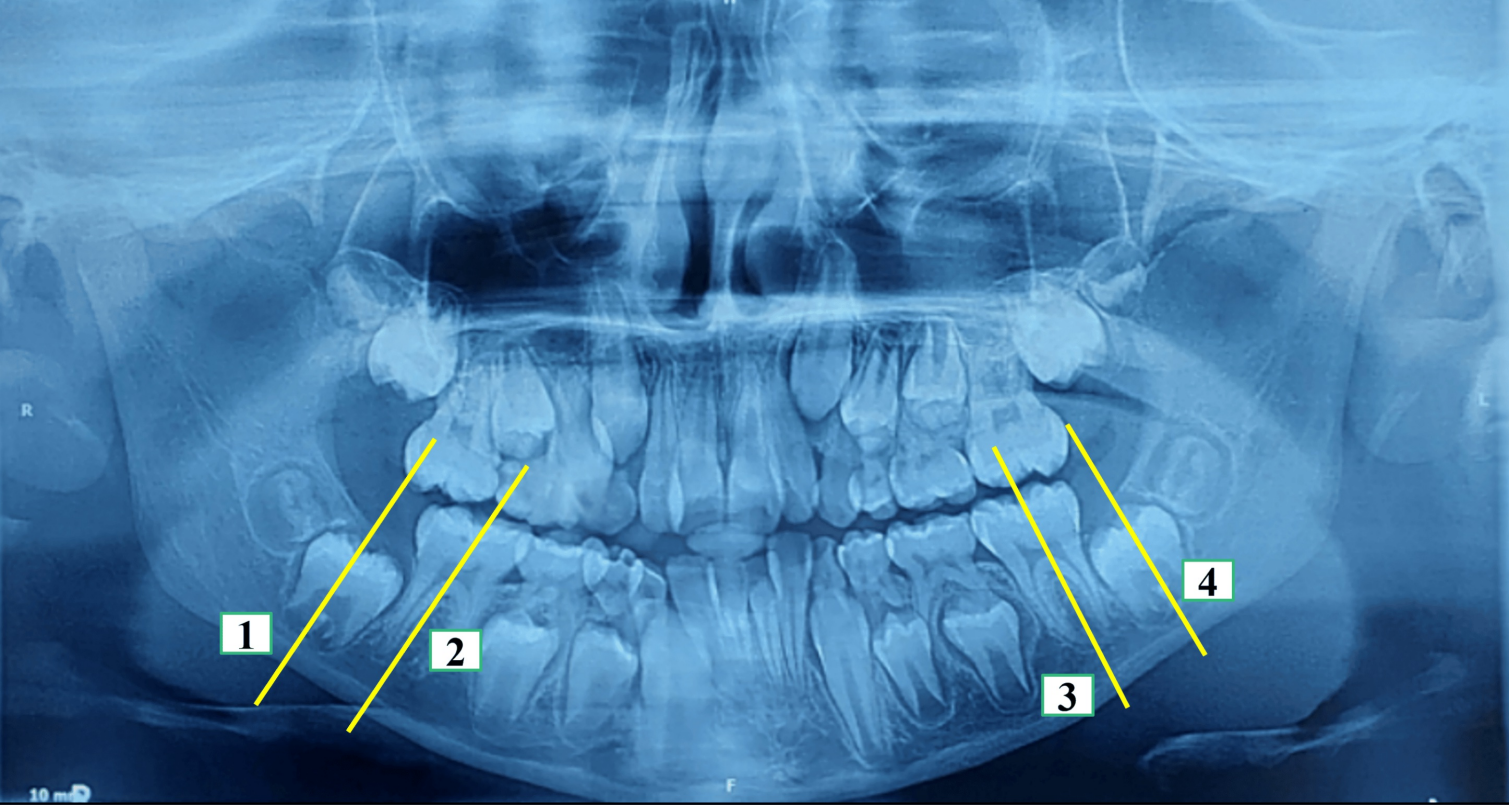
Pretreatment intermolar angle between the first and second permanent mandibular molars was found to be approximately zero (lines 1, 2, 3, and 4 represent the long axis of the first and second mandibular permanent molars)

However, preservation of leeway space aligns anterior crowding, but it is not complication-free in the arches with end-on molar relation, as by not allowing the first permanent molar to shift mesially, it encroaches on the second and third molar spaces later [10]. Our case had bilateral Class I molar relation; thus, space gain was not at the expense of posterior molars. The pretreatment intermolar angulation between the first and second permanent mandibular molars was found to be approximately zero (Figure 13), indicating a lower probability of second molar impaction, as suggested by Sonis and Ackerman [18].

However, the patient is kept on follow-ups to verify this prediction and was also made aware that, in view of evolutionary arch length reduction, third molars might need extraction.

Mixed dentition analysis plays a critical role in planning effective, evidence-based treatment for crowding during the transitional phase of dentition. By quantifying TSALD and evaluating ISD and BSD, clinicians can accurately determine whether space is sufficient for the proper alignment of erupting permanent teeth. A data-driven understanding of space availability not only helps localize the area of discrepancy but also tailors the treatment to individual growth patterns, ensuring more stable and efficient long-term outcomes. Usually, extraction-based approaches are utilized for the management of these types of severely crowded cases presenting with constricted arch, but in this case, thorough evaluation of both static and functional occlusion, in conjunction with the model analysis, enabled the development of an effective non-extraction interceptive orthodontic treatment plan.

## Conclusions

This case report illustrates that comprehensive analytical interceptive orthodontic therapy for developing malocclusion, in mixed dentition, when provided in well-defined stages through the control of space supervision can be highly successful. Identification and remediation of crowding during the mixed dentition stage allow for early treatment, which often prevents extensive interventions at a later stage. By controlling the trajectory of permanent teeth and preserving or utilizing leeway space, teeth can be guided to their intended positions, reducing the severity of malocclusion. This stepwise approach, along with thorough knowledge of developing occlusion, not only maximizes functional and esthetic outcomes but also maximizes oral hygiene and reduces the risk for caries, periodontal diseases, and traumatic dental injuries. Our case describes the method to change severe crowding to mimic the developing occlusion patterns as in ideal borderline cases by simple maneuvers through thorough examinations and investigations. The analytical treatment plan changed the extraction case to a non-extraction one, facilitating arch dimensions and other oral functions. 
